# Supplementary material for: Early life adversity decreases pre-adolescent fear expression by accelerating amygdala PV cell development
Source: eLife. 2020 Jul 21;9:e55263. doi: 10.7554/eLife.55263 (PMC7413666; doi:10.7554/eLife.55263)
Supplement: Transparent reporting form [file elife-55263-transrepform.pdf]

eLIFE
1st Floor
24 Hills Road
Cambridge CB2 1JP, UK
P 01223 855340
W elifesciences.org
T @elife

eLife's transparent reporting form

We encourage authors to provide detailed information *within their submission* to facilitate the interpretation and replication of experiments. Authors can upload supporting documentation to indicate the use of appropriate reporting guidelines for health-related research (see EQUATOR Network), life science research (see the BioSharing Information Resource), or the ARRIVE guidelines for reporting work involving animal research. Where applicable, authors should refer to any relevant reporting standards documents in this form.

If you have any questions, please consult our Journal Policies and/or contact us: editorial@elifesciences.org.

Sample-size estimation

- You should state whether an appropriate sample size was computed when the study was being designed
- You should state the statistical method of sample size computation and any required assumptions
- If no explicit power analysis was used, you should describe how you decided what sample (replicate) size (number) to use

Please outline where this information can be found within the submission (e.g., sections or figure legends), or explain why this information doesn't apply to your submission:

Sample sizes were based on observed differences and variance in preliminary studies, replication experiments, and sample sizes used for similar studies that had been previously published.

Replicates

- You should report how often each experiment was performed
- You should include a definition of biological versus technical replication
- The data obtained should be provided and sufficient information should be provided to indicate the number of independent biological and/or technical replicates
- If you encountered any outliers, you should describe how these were handled
- Criteria for exclusion/inclusion of data should be clearly stated
- High-throughput sequence data should be uploaded before submission, with a private link for reviewers provided (these are available from both GEO and ArrayExpress)

Please outline where this information can be found within the submission (e.g., sections or figure legends), or explain why this information doesn't apply to your submission:

A description of experimental design can be found in the methods section. The main behavioral results (decreased freezing at PND 22) was replicated across 3 independent experiments. For all experiments multiple cohorts were used, and the total number of mice (n) for each group is provided within the figure legends. No outliers needed to be removed from the datasets. For optogenetic, and cholera toxin B experiments, exclusion/inclusion was based on placement of optic fiber or injection respectively.

eLife Sciences Publications, Ltd is a limited liability non-profit non-stock corporation incorporated in the State of Delaware, USA, with company number 5030732, and is registered in the UK with company number FC030576 and branch number BR015634 at the address 1st Floor, 24 Hills Road, Cambridge CB2 1JP | August 2014

Statistical reporting

- Statistical analysis methods should be described and justified
- Raw data should be presented in figures whenever informative to do so (typically when N per group is less than 10)
- For each experiment, you should identify the statistical tests used, exact values of N, definitions of center, methods of multiple test correction, and dispersion and precision measures (e.g., mean, median, SD, SEM, confidence intervals; and, for the major substantive results, a measure of effect size (e.g., Pearson's r, Cohen's d)
- Report exact p-values wherever possible alongside the summary statistics and 95% confidence intervals. These should be reported for all key questions and not only when the p-value is less than 0.05.

Please outline where this information can be found within the submission (e.g., sections or figure legends), or explain why this information doesn't apply to your submission:

Statistical tests and their values are provided in the figure legends. In general, we focused on analyzing the effects of rearing condition at each age tested, instead of across time. This decision was in part based on the difficulty of simultaneously running all ages, sexes and groups in assays such as western blot and immunohistochemistry. However, in behavioral experiments, where large number of animals could be run across ages, 3-way ANOVAS which included effects of age, sex, and rearing condition were conducted.

(For large datasets, or papers with a very large number of statistical tests, you may upload a single table file with tests, Ns, etc., with reference to sections in the manuscript.)

Group allocation

- Indicate how samples were allocated into experimental groups (in the case of clinical studies, please specify allocation to treatment method); if randomization was used, please also state if restricted randomization was applied
- Indicate if masking was used during group allocation, data collection and/or data analysis

Please outline where this information can be found within the submission (e.g., sections or figure legends), or explain why this information doesn't apply to your submission:

Litters of mice were randomly assigned to limited bedding or control bedding rearing conditions. All mice of a given litter were thus designated at limited bedding (experimental) or control rearing (control) mice. When possible, automated software was used to eliminate experimenter bias. Any exception to automated analysis is detailed in the figure legend or within the methods section. Within the optogenetic experiments all mice in a litter were implanted, with genotyping for Halo+ status being conducted after the experiment had been completed. This assured no bias in the treatment of Halo+ versus Halo- mice.

Additional data files ("source data")

- We encourage you to upload relevant additional data files, such as numerical data that are represented as a graph in a figure, or as a summary table
- Where provided, these should be in the most useful format, and they can be uploaded as "Source data" files linked to a main figure or table
- Include model definition files including the full list of parameters used
- Include code used for data analysis (e.g., R, MatLab)

eLife Sciences Publications, Ltd is a limited liability non-profit non-stock corporation incorporated in the State of Delaware, USA, with company number 5030732, and is registered in the UK with company number FC030576 and branch number BR015634 at the address 1st Floor, 24 Hills Road, Cambridge CB2 1JP | August 2014

- Avoid stating that data files are "available upon request"

Please indicate the figures or tables for which source data files have been provided:

Source data files for all figures have been uploaded to the Brown Library data archive. The DOI for the raw data is: https://doi.org/10.26300/9krc-h052
